# Cortical-wide functional correlations are associated with stress-induced cardiac dysfunctions in individual rats

**DOI:** 10.1038/s41598-019-47171-y

**Published:** 2019-07-22

**Authors:** Ryota Nakayama, Yuji Ikegaya, Takuya Sasaki

**Affiliations:** 1 Laboratory of Chemical Pharmacology, Graduate School of Pharmaceutical Sciences, The University of Tokyo, 7-3-1 Hongo Bunkyo-ku, Tokyo, 113-0033 Japan; 2 Center for Information and Neural Networks, Suita City, Osaka, 565-0871 Japan; 3 Precursory Research for Embryonic Science and Technology, Japan Science and Technology Agency, 4-1-8 Honcho, Kawaguchi, Saitama 332-0012 Japan

**Keywords:** Stress and resilience, Neuronal physiology

## Abstract

Mental stress-induced biological responses considerably differ across animals, which may be explained by intrinsic brain activity patterns. To address this hypothesis, we recorded local field potential signals from six cortical areas, electrocardiograms, and electromyograms from freely moving rats. Based on their stress-induced changes in cardiac signals, individual defeated rats were classified into stress susceptible and resilient groups. Rats with lower correlations in theta power across wide ranges of cortical regions before the stress challenge had higher probability to be stress-susceptible rats as defined based on the irregularity of heartbeat signals. A combination of principal component analysis and the support vector machine algorithm revealed that functional connections across cortical regions could be predictive factors accounting for individual differences in future stress susceptibility. These results suggest that individual differences in cortical activity may be a mechanism that causes abnormal activity of peripheral organs in response to mental stress episodes. This evidence will advance the understanding of the neurophysiological correlates of mind-body associations during mental stress exposure.

## Introduction

Stressful experiences trigger a series of complex biological responses in the whole body. Although many early studies revealed stress-induced biological factors at the molecular and cellular levels^1,2^, relatively few systemic physiological studies provided insights into stress responses, especially for functional interactions across the central and peripheral organs. At the macroscopic level, a number of behavioral studies have assessed how stressful experiences alter subsequent behavioral patterns, such as changes in anxiety-like and depressive-like behavior^3–5^. To connect existing evidence from the molecular to the behavioral levels, further studies are required to reveal the effects of stress loads on ongoing physiological organ dynamics.

Notably, stress-induced responses considerably differ across individual animals. A subset of animals exhibit large stress-induced reactions (so-called stress susceptibility), whereas other animals show no apparent changes (so-called stress resiliency)^6–8^. Since an initial step in most biological reactions induced by stress, especially mental stress, is information processing in the brain, the different degree of stress susceptibility may be accounted for by brain activity patterns. To support this hypothesis, recent studies have demonstrated that correlational activity across multiple brain regions before experiencing stressful episodes significantly differs and is associated with subsequent stress-induced responses, such as depression-like behavior^9,10^.

To address the hypothesis that brain activity is a crucial determinant of stress susceptibility, we performed multisite recordings of local field potential (LFP) signals from rat cortical regions. The rats were exposed to social defeat (SD) stress, which was shown to evoke profound changes in rat’s behavioral patterns, such as increased anxiety-like and depressive-like behavior^6–8,11^. Importantly, the stress loads applied to the rats in these studies yielded pronounced individual differences in their behavioral results. In this study, to quantify detailed stress responses in addition to behavioral phenotypes, we integrated recordings of electrocardiogram (ECG) signals, which represented the cardiac rhythm, and electromyogram (EMG) signals, which represented awake-related muscle contraction, into the recording system for our cortical-wide LFP recordings^12–14^. Based on the classification of stress-induced peripheral physiological activity, we analyzed whether multidimensional cortical LFP signals before experiencing SD stress were related to subsequent stress susceptibility.

## Results

### Simultaneous recording of ECG and EMG signals with cortical LFP signals

**Figure 1:**
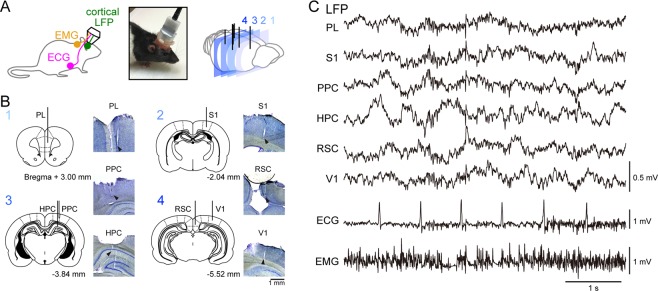
Simultaneous electrophysiological recordings of cortical LFP, ECG, and EMG signals from a freely moving rat. (**A**) (Left) A schematic illustration of the recording system. (Middle) A picture of a protected recording device that is tolerable to physical attacks from the other rat. (Right) The brain was coronally sectioned and magnified in B. (**B**) Brain coronal sections showing electrode positions and histological confirmation of the electrode locations (arrows). The numbers in the upper left correspond to the brain sections in A. (**C**) Representative electrophysiological traces of the cortical LFP, ECG and EMG signals.

We recently established a recording system that enabled simultaneous monitoring of LFPs, ECG, and EMG in a freely moving rat using a custom-made plastic device (Fig. 1A). In this study, we created a plastic cover to mount on the rat’s head and protect the recording device against physical attacks from the other rat (Fig. 1A, middle). For the LFP recordings, six cortical regions were targeted, including the prelimbic cortex (PL), primary somatosensory cortex (S1), posterior parietal cortex (PPC), hippocampus (HPC), retrosplenial cortex (RSC), and primary visual cortex (V1) (Fig. 1B), and verified by histological inspections. These brain areas were selected so that they cover the wide range of cortex from anterior to posterior and from medial and lateral parts as much as possible. Figure 1C shows representative simultaneous recordings of all bioelectrical signals from the central and peripheral organs in a freely moving rat.

### Classification of stress susceptibility based on heartbeat changes

**Figure 2:**
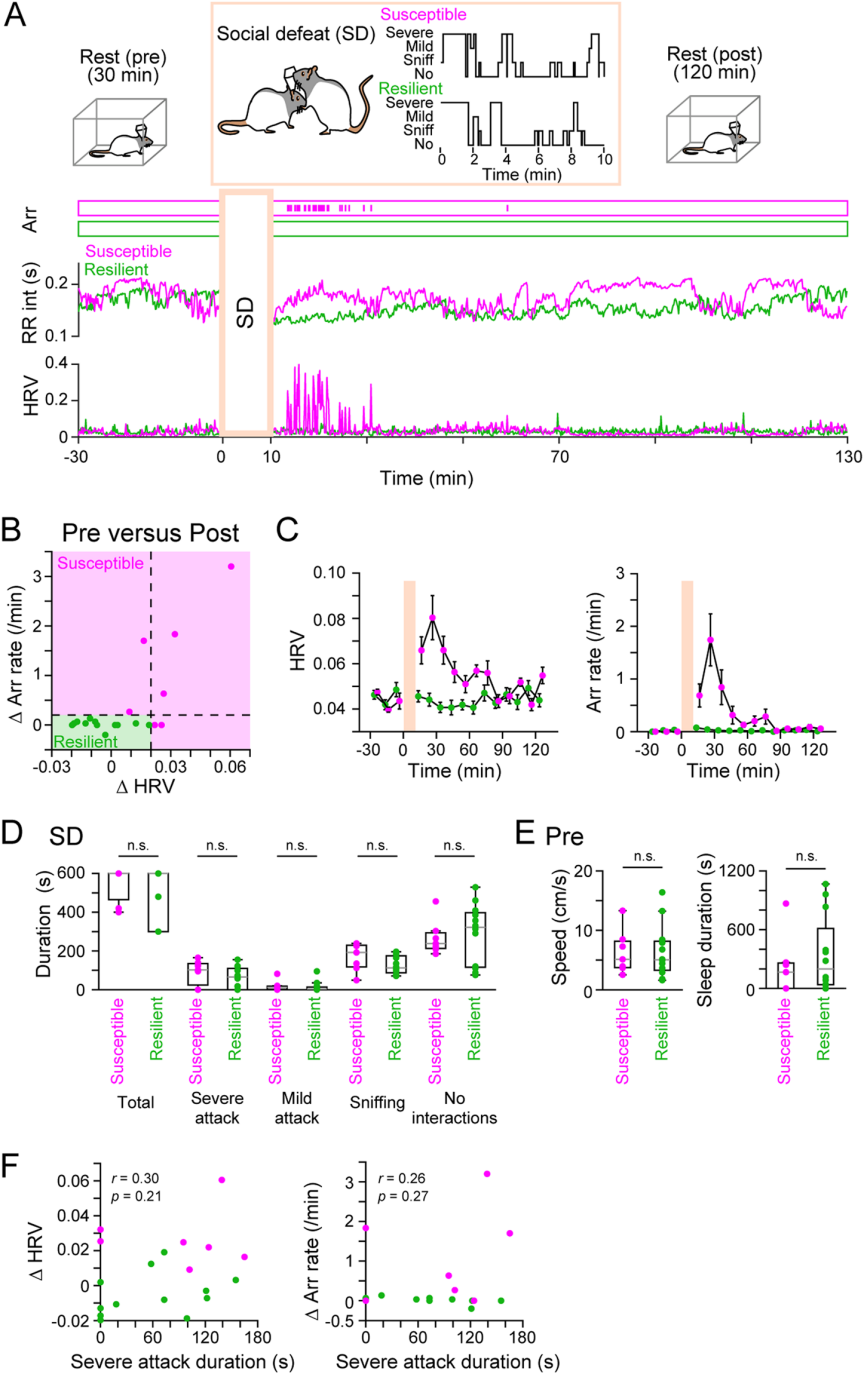
Changes in cardiac signals in response to acute SD stress. (**A**) (Top) After staying in a rest box for 30 min (pre session), a recorded rat was placed in an open field and loaded with SD stress from a resident rat for up to 10 min (SD session). After the SD session, the defeated rat was placed back in the same box (post session) (Bottom). Representative time changes in cardiac signals showing the time of arrhythmia (arr; upper ticks), instantaneous RR intervals, and HRV in a resilient (green) and susceptible (magenta) rat. The top inset within the orange box shows the physical attack patterns during the SD session (severe, severe attack; mild, mild attack; sniff, sniffing; no, no interactions). (**B**) A summary plot of changes in the HRV and arrhythmia (arr) rates after SD stress (30 min). Each dot represents one rat. Susceptible rats were defined if their Δarrhythmia (Δarr) rate exceeded 0.2/min or ΔHRV exceeded 0.02. These factors have a significant positive correlation (*r* = 0.73, *p* = 3.5 × 10^−4^). (**C**) Average changes in the HRV and arrhythmia rates over time for each rat type. (**D**) No significant differences were found in the total duration of the SD session and the duration of the four behavioral patterns between the susceptible and resilient groups. Each dot represents one rat. (**E**) No significant differences in the moving speed and sleep duration in the pre rest session were observed between the susceptible and resilient groups. (**F**) No significant correlations were found between severe attack duration and ΔHRV (left) or Δarr rate (right). Each dot represents one rat.

In total, 19 rats were recorded and loaded with SD stress by exposing them to a resident rat for up to 10 min (termed a SD session). Before and after the SD session, the rat was placed in the rest box for 30 min and 120 min (termed the pre and post sessions, respectively), during which time the electrophysiological recordings of the LFP, ECG, and EMG signals were continuously monitored (Fig. 2A, top). The signals obtained from the SD session were not analyzed because these signals included a massive amount of electrical noise due to intense physical movement. During the pre and post session recordings, the heartbeat time was detected to quantify stress-induced changes in the irregularity of cardiac cycles. As demonstrated by a previous study^15^, some rats exposed to acute SD stress exhibited unstable cardiac cycles, including arrhythmia and increased HRV, in the post session. A susceptible rat was defined as a rat that showed either one of following criteria computed between the 10 min pre and 30 min post session: Δarrhythmia rate more than 0.2/min or ΔHRV more than 0.02. The classification results from all 19 defeated rats tested are summarized in Fig. 2B, showing a significant positive correlation between the two factors (*r* = 0.73, *p* = 3.5 × 10^−4^). Figure 2A (bottom) shows representative susceptible (magenta) and resilient (green) rats. As shown in Fig. 2C, irregular cardiac responses specifically occurred during the first 30 min of the post session in the susceptible group. The total duration of the SD and the duration of all attack patterns (i.e., severe attack, mild attack, sniffing, and no interactions) did not significantly differ between the susceptible and resilient groups (Fig. 2D; total duration: t_17_ = 0.91, *p* > 0.99; severe attack: t_17_ = 1.0, *p* > 0.99; mild attack: t_17 = _0.31, *p* > 0.99; sniffing: t_17_ = 1.6, *p* = 0.65; no interactions, t_17_ = 0.32, *p* > 0.99, Student’s *t*-test with Bonferroni correction). These results suggest that the variation in stress susceptibility is not simply due to individual differences in the intensity and duration of the physical attacks from the resident rats. The running speed and the sleep duration in the pre session did not significantly differ between the two groups (Fig. 2E; speed: t_17_ = 0.054, *p* = 0.95; sleep duration: t_17_ = 0.74, *p* = 0.47, Student’s *t*-test). These results suggest that baseline locomotor activity before applying stress loads is not likely to be a determinant of stress susceptibility. In addition, we found no significant correlation between the duration of severe attack intensity and ΔHRV (*r* = 0.30, *p* = 0.21) or Δarrhythmia rate (*r* = 0.28, *p* = 0.27) (Fig. 2F), demonstrating that the variability of attack intensity during the SD session alone did not account for resultant stress susceptibility.

### LFP signals at single cortical regions do not account for stress susceptibility

**Figure 3:**
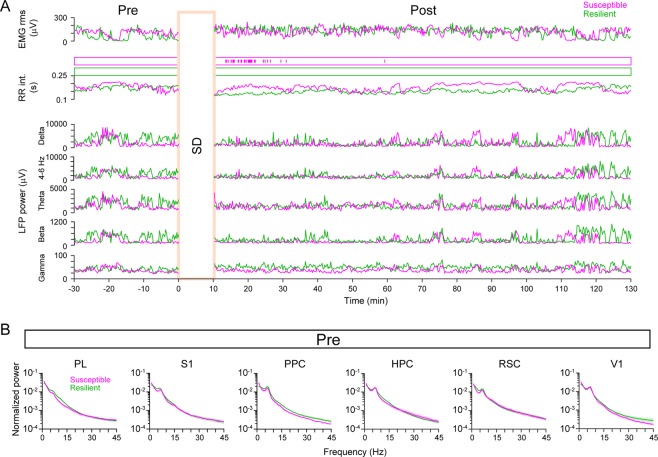
Cortical LFP power of individual cortical areas in the pre rest session did not differ between the stress susceptible and resilient groups. (**A**) (From top to bottom) Time changes in the rms of the EMG signals, time of arrhythmia, instantaneous RR intervals, and hippocampal LFP power at the delta, theta, and gamma bands in representative resilient and susceptible rats. (**B**) No significant differences were observed in the average LFP power spectrum in the PL, HPC, PPC, RSC, S1 and V1 between the stress susceptible and resilient groups. Shaded areas represent SEMs.

Next, we tested the possibility that cortical activity states during rest in the pre session might be associated with subsequent stress susceptibility. For each cortical region, LFP power in the pre session was computed by Fourier transformation. Figure 3A shows typical temporal changes in the root-mean-square (rms) of the EMG, time of arrhythmia, instantaneous R-R intervals, and hippocampal LFP power at the delta (1–4 Hz), 4–6 Hz (a middle band between delta and theta bands), theta (6–10 Hz), beta (10–25 Hz), and gamma (25–45 Hz) bands. For all cortical regions tested, we found no significant differences in the LFP power at all frequency bands from 1 to 45 Hz in the pre session between the susceptible and resilient groups (Fig. 3B; *q* > 0.05 at all frequency bands, FDR corrected). These results demonstrate that LFP power in single cortical regions alone cannot account for subsequent susceptibility against SD stress.

### Correlational LFP power changes across multiple cortical regions are related to subsequent stress susceptibility

**Figure 4:**
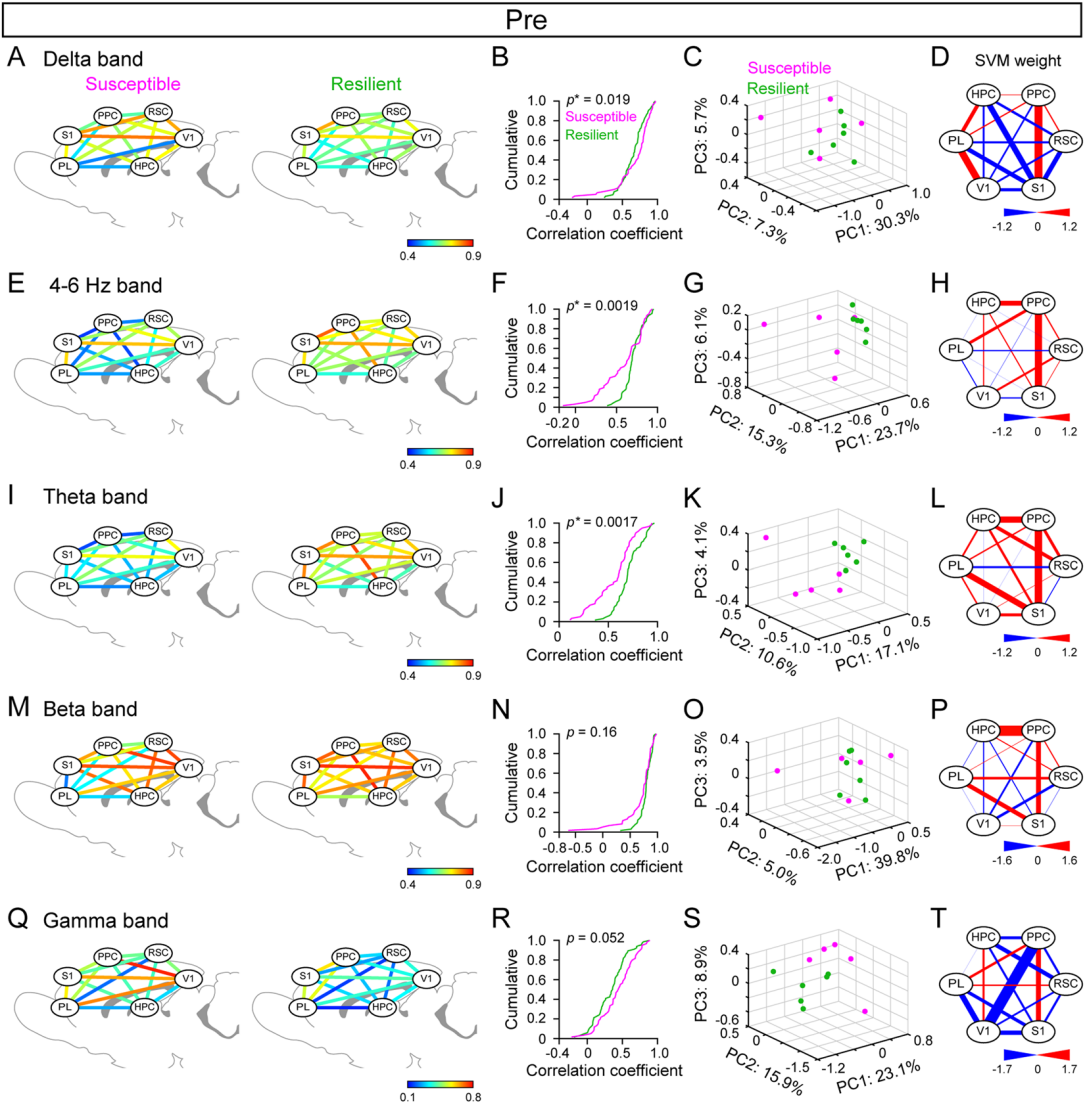
Differences in LFP power correlations across wide-cortical areas in the pre rest session between the stress susceptible and resilient groups. (**A**) Color-coded maps showing the average LFP delta power correlations computed between individual pairs of cortical areas. (**B**) The cumulative distribution of the LFP delta power correlations in 60 and 66 pairs of cortical areas obtained from 5 susceptible and 6 resilient rats, respectively. The statistical result was provided by the Kolmogorov-Smirnov test. (**C**) A three-dimensional plot of the three principal components decomposed from all LFP delta power correlations obtained from individual rats. Each dot represents one rat (magenta, susceptible; green, resilient). Contribution ratios for individual dimensions are described in individual axes. The loss percentage computed from the SVM algorithm with a cross validation method is shown at the top on the right. (**D**) Weight of each cortical region pair defined by the SVM algorithm. (**E**–**H**) Same as **A**–**D** but for 4–6 Hz band. (**I**–**L**) Same as **A**–**D** but for the theta band. (**M**–**P**) Same as **A**–**D** but for the beta band. (**Q**–**T**) Same as **A**–**D** but for the gamma band.

Then, we examined whether regional correlations of LFP power changes in the cortical network (so-called functional connectivity) might be associated with SD stress susceptibility. To obtain an overview of functional cortical organizations, first we computed correlation coefficients of LFP power changes in the 30-min pre session (*n* = 5 susceptible and 6 resilient rats). The averaged correlation coefficients obtained from all cortical region pairs (_6_C_2_ = 15) within each group are summarized in Fig. 4A,E,I,M, and 4Q for the delta, 4–6 Hz, theta, beta, and gamma bands, respectively. The cumulative distributions of these coefficients revealed significant differences in regional correlations between the susceptible and resilient groups at the lower ( < 10 Hz) bands (Fig. 4B,F,J; *n* = 60 and 66 pairs of cortical areas from 5 susceptible and 6 resilient rats; delta: *D*_*max*_ = 0.27, *p* = 0.019; 4–6 Hz: *D*_*max*_ = 0.32, *p* = 0.0019; theta: *D*_*max*_ = 0.33, *p* = 0.0017, Kolmogorov-Smirnov test) but not the higher ( > 10 Hz) bands (Fig. 4N,R; beta: *D*_*max*_ = 0.20, *p* = 0.16; gamma: *D*_*max*_ = 0.23, *p* = 0.052, Kolmogorov-Smirnov test). These results demonstrate that the resilient rats show have higher temporal correlations of LFP power at the theta band across cortical-wide regions prior to loading SD stress, compared with the susceptible rats. To further examine whether such correlational changes in LFP power is associated with the strength of stress susceptibility, we computed correlations between averaged correlation coefficients of LFP power changes at each frequency band for each animal and its or ΔHRV. However, this analysis failed to detect significant correlations except for the gamma band (Δarrhythmia rate, delta: *r* = 0.51, *p* = 0.11; 4–6 Hz: *r* = 0.62, *p* = 0.068; theta: *r* = −0.17, *p* = 0.62; beta: *r* = −0.58, *p* = 0.059; gamma: *r* = 0.64, *p* = 0.034; ΔHRV, delta: *r* = 0.46, *p* = 0.15; 4–6 Hz: *r* = 0.051, *p* = 0.88; theta: *r* = −0.27, *p* = 0.42; beta: *r* = −0.50, *p* = 0.12; gamma: *r* = 0.63, *p* = 0.039), showing that the strength of stress-induced cardiac dysfunction is not simply accounted for by correlational LFP power changes across brain regions alone.

Based on these statistical results, we next addressed population analyses by applying the principal component analysis (PCA) and the support vector machine (SVM) algorithm to the same correlation coefficient datasets. First, the correlation coefficients of all cortical region pairs for each frequency band were decomposed by the PCA, and their first three principal components were plotted in a three-dimensional space (Fig. 4C,G,K,O,S). Next, a linear SVM, which is a supervised machine learning algorithm, was applied to the same datasets to determine the cutoff line that best separated the susceptible and resilient groups. Figure 4D,H,L,P,T shows all weights for individual cortical region pairs to define by the SVM analysis. The failure percentages calculated from the leave-one-out cross validation analysis were 63.7%, 9.1%, 9.1%, 18.2%, and 9.1%, for the delta, 4–6 Hz, theta, beta, and gamma bands, respectively. In other words, the SD stress susceptibility of more than 90% of the rats could be correctly predicted from cortical regional LFP power correlations, especially for > 4 Hz band. These results further support that functional connectivity across cortical-wide regions at resting states is a possible determinant of a rat’s susceptibility in response to subsequent stress episodes.

### Post-stress LFP power changes in stress susceptible and resilient rats

**Figure 5:**
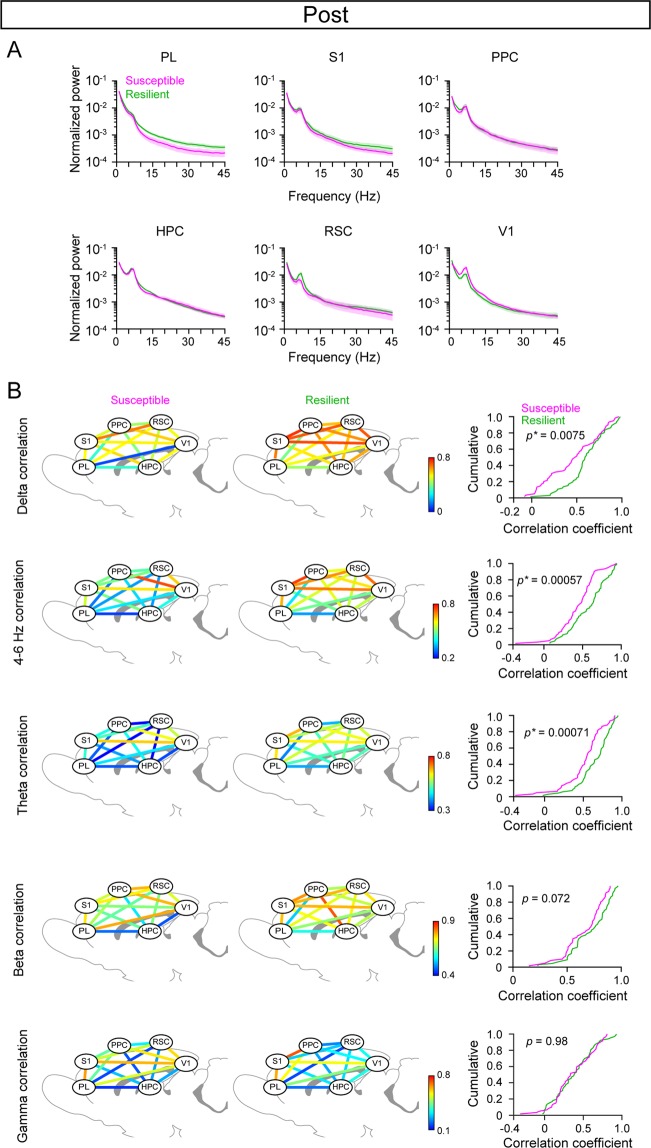
Changes in LFP power in individual cortical regions after experiencing SD stress. (**A**) Same as Fig. 3B but for the post session. No significant differences were observed in the LFP power spectra of individual cortical areas. (**B**) Same as Fig. 4 but computed for the post session. A significant difference was found in the cumulative distribution at the delta to theta bands between the stress susceptible and resilient groups.

The results described above focused on how cortical LFP power before applying stress was related to stress susceptibility. Next, we examined how the cortical LFP power changed after SD stress using the same analyses shown in Fig. 3B (Fig. 5). Fourier transformation detected no differences in the LFP power in individual cortical areas during the 30-min periods over a frequency band of 1–45 Hz between the resilient and susceptible groups (Fig. 5A; *q* > 0.05 at all frequency bands, FDR corrected). Cumulative distribution analyses revealed that the resilient group showed significantly higher regional correlation coefficients at the delta and theta band than the susceptible group (Fig. 5B; delta: *D*_*max*_ = 0.29, *p* = 0.0075; 4–6 Hz: *D*_*max*_ = 0.35, *p* = 0.00057; theta: *D*_*max*_ = 0.35, *p* = 0.00071; beta: *D*_*max*_ = 0.22, *p* = 0.072; gamma: *D*_*max*_ = 0.080, *p* = 0.98).

## Discussion

In this study, we obtained LFP signals simultaneously from six cortical regions together with ECG and EMG signals from a single rat exposed to acute SD stress. ECG recordings demonstrated that rats subjected to SD stress exhibited heterogeneous responses in cardiac signals, resulting in stress susceptible and resilient groups. A multidimensional analysis with the support vector machine showed that correlational power changes across cortical regions were predictive for such individual differences in stress susceptibility.

In the majority of stress studies, stress susceptibility has been quantified by changes in behavioral patterns, such as decreased social interactions and increased anxiety-like behavior in the social interaction and open field tests, respectively^4–8^. However, it is impossible to perfectly estimate animals’ mental states from specific behavioral signs alone. On the other hand, it has been well known that stress loads cause abnormal changes in peripheral activity patterns such as heartbeat signals and respiratory signals in both animal models and human patients, possibly via abnormal changes in autonomic transmission. Therefore, animal’s mental states can be more accurately evaluated by measuring autonomic physiological responses of internal organs. Based on this assumption, we recorded ECG and EMG signals during pre- and post-stress periods, allowing an ECG-based classification of the stress susceptible and resilient groups. These analyses confirmed that peripheral physiological signals can be an useful parameter to estimate animals’ psychiatric states in addition to simple behavioral patterns.

The rodent brain forms functional network organizations at a resting state (termed the default mode-like network)^16,17^ similar to the human brain. We showed that LFP power arising from individual cortical regions in the pre session, which was a period that possibly included a resting state, was not significantly different between the stress susceptible and resilient groups. Conversely, regional LFP power correlations in the pre session showed significant differences between the two groups. These results are consistent with previous studies showing that functional connectivity across certain brain regions, rather than activity levels of single brain areas, is crucial for accounting for mental disorders in rodents^9,10,18,19^ and humans^20,21^. Moreover, we demonstrated that individual differences in such functional organizations of cortical-wide networks observed before loading stress could predict the heterogeneity of future stress susceptibility. These insights are consistent with recent studies that have shown that monitoring cortical neuronal activity is a useful method to predict susceptibility to mood disorders, such as major depression disorder,^10^ and the therapeutic effects of drugs against mood disorders^20^.

Cortical LFP activity reflects populational neuronal activity in the brain and is linked with a variety of information processing, including cognition, learning and memory^22^. In the neocortex, generally delta LFP oscillations are larger during low arousal states, such as sleep and quiet states^23,24^. Our results showed that rats with higher theta power correlations, which are generally more dominant during movement or memory acquisition^25,26^, showed stable heartbeat signals against SD stress. These results suggest that rats showing stronger movement-related cortical activity, possibly representing higher arousal states, are more resilient to stress loads.

In traditional views, physiological organ activity has been described by the subdivisions of research area describing a single organ or single function, such as one brain region, a cardiac system, and a respiratory system. However, all organs continuously transmit information each other. To address this issue, a systemic viewpoint is further required to examine interactions across multiple organs. In this view, our study provides a typical example implying that peripheral dysfunctions may arise from cortical functional organizations with highlighting the importance of large-scale physiological data analyses in which both the central and peripheral organs are combined. In future studies, these research ideas will help deepen our understanding of the brain-body associations in health and disease.

## Materials and Methods

### Ethical approval

This study was carried out in accordance with NIH guidelines for the care and use of rats. The protocol was approved by the Experimental Animal Ethics Committee of the University of Tokyo (approval number: P29–7).

### Animals

A total of 19 male Long-Evans rats (10–15 weeks old) with preoperative weights of 300–350 g were recorded and exposed to SD stress in this study. To apply SD stress to these rats, an additional 2 male Long-Evans rats (more than 4 months old) with weights of 450–550 g and 2 female Long-Evans rats (more than 4 months old) with weights of 300–400 g were used as the resident rats. The rats were housed individually and maintained on a 12-h light/12-h dark schedule with lights off at 7:00 AM unless otherwise specified. All rats were purchased from SLC (Shizuoka, Japan). After at least 1 week of adaptation to the laboratory, the rats underwent surgery.

### Surgery

Surgery was performed to implant electrodes for recording ECG, EMG and LFP signals from a single rat. The detailed surgical procedures have been described elsewhere^12–14^. Briefly, before surgery, an electrode comprising a core body and a custom-made electrical interface board (EIB) accommodating at most 6 LFP channels, 2 ECG channels, 2 EMG channels, and 2 ground/reference channels was assembled. For the surgery, the rats were anesthetized with 1–2% isoflurane gas in air. For 19 rats, two incisions (∼1 cm) were made on both sides of the upper chest, and 2 stainless steel ECG electrodes with a tip dimeter of 0.147 mm diameter (AS633, Cooner Wire Company) in which the PTFE coating at the tip was peeled off at a length of ~5.0 mm were sutured to the tissue underneath the skin of the upper chest. Then, the rat was fixed in a stereotaxic instrument with two ear bars and a nose clamp. A midline incision was made from the area between the eyes to the incised neck area, and 2 stainless steel EMG electrodes with a tip dimeter of 0.147 mm diameter (AS633, Cooner Wire Company) in which the PTFE coating at the tip was peeled off at a length of ~5.0 mm were sutured to the dorsal neck muscles. For 11 of the 19 rats, LFP electrodes were implanted into the brain. Circular craniotomies 0.9 mm in diameter were made using a high-speed drill at the following coordinates: 3.0 mm anterior and 0.5 mm lateral to the bregma for the prelimbic cortex, 2.0 mm posterior and 2.8 mm lateral to the bregma for the primary somatosensory cortex, 3.8 mm posterior and 2.8 mm lateral to the bregma for the posterior parietal cortex and the hippocampus, 5.5 mm posterior and 0.5 mm lateral to the bregma for the retrosplenial cortex, and 5.5 mm posterior and 4.0 mm lateral to the bregma for the primary visual cortex. For the prelimbic cortex and hippocampus, electrodes were implanted at a depth of 2.5 mm. For the retrosplenial cortex, an electrode was implanted at a depth of 1.5 mm. For the primary somatosensory cortex, visual cortex, and posterior parietal cortex, electrodes were implanted at a depth of 1.2 mm. For the cerebellum, stainless steel screws were implanted on the skull attached to the brain surface to serve as ground/reference electrodes. The open edges of all electrodes were soldered to the corresponding channels on the EIB. All wires and the electrode assembly were secured to the skull using dental cement. After completing all surgical procedures, the anesthesia was removed, and the rats were allowed to awaken from the anesthesia spontaneously. Following surgery, each rat was housed in a transparent Plexiglas cage with free access to water and food.

### Electrophysiological recording

At least 7 days after surgery, each rat with implanted electrodes was connected to recording equipment via Cereplex M (Blackrock), which is a digitally programmable amplifier, close to the rat’s head. The headstage output was conducted via a lightweight multiwire tether and a commutator to the Cereplex Direct recording system (Blackrock), which is a data acquisition system. LFP recordings were sampled at 2 kHz and filtered between 1 and 500 Hz.

### Acute SD stress

A recording day for each rat consisted of three sessions: a 30-min pre session in a rest box (24 cm × 40 cm × 35 cm), an up to 10 min SD session in an open field (50 cm × 50 cm × 60 cm), and a 120-min post session in the same rest box. Acute SD stress was applied to the recorded rat similar to rat resident-intruder tests^15,27^. To prepare an experimental environment for the SD session, a male and a female rat were housed as resident rats in the open field at least one hour per day for 7 days before the SD session. When starting the SD session, the female resident rat was removed from the field, and the recorded rat was placed as an intruder rat into the open field. During the SD session, the intruder recorded rat was physically attacked by the resident male rat for 10 min. The SD session was immediately terminated if the intruder rat had a wound and bleeding due to the attack. The resident male rats were selected for their attack latencies shorter than 60 s when they were exposed to several rats that were not used for the subsequent experiments. The physical attack patterns of the resident rats were categorized into four types: (i) severe attack, including biting and chasing, (ii) mild attack, including mounting and touching, (iii) sniffing, and (iv) no interactions. The rat’s moment-to-moment position was tracked using a video camera attached to the ceiling with a frame rate of 3 Hz.

### Histological analysis to confirm electrode locations

The rats were overdosed with urethane/α-chloralose, perfused intracardially with 4% paraformaldehyde (PFA) in phosphate-buffered saline (PBS; pH 7.4) and then decapitated. After dissection, the brains were fixed overnight in 4% PFA and then equilibrated with 30% sucrose in PBS. Frozen coronal sections (100 µm) were cut using a microtome, and serial sections were mounted and processed for cresyl violet staining. For cresyl violet staining, the slices were rinsed in water, counterstained with cresyl violet, and coverslipped with Permount. The positions of all electrodes were confirmed by identifying electrode tracks in the brain slices.

### Analysis of electrophysiological traces

All analyses were performed using Matlab (Mathworks). ECG traces were bandpass filtered at 20–200 Hz, and beat-to-beat intervals (R-R interval) were calculated from the timestamp of the R-wave peaks. The root-mean-square (rms) of the EMG signals was computed in each bin with a bin size of 500 ms, which represented the absolute changes in EMG amplitude relative to the average. The LFP traces were downsampled to 200 Hz, and the LFP power was calculated by fast Fourier transform in each 5-s time window. Based on the time changes in LFP power, Pearson’s correlation coefficient was computed from a pair of cortical regions. Although our recordings targeted six cortical regions, which yielded complete datasets, one region was missed in 3 susceptible rats and 3 resilient rat, and two regions were missed in 1 resilient rat. In these rats, the data points at the missed areas were filled with average values of the corresponding areas computed from the other animals classified in the same group. The instantaneous speed of each frame was calculated based on the distance traveled within a frame (~333 ms). A sleep period was detected when the rms amplitude of the EMG was less than the mean–SD and lasted for 10 s, where the mean and SD were the average and standard deviation of the top 20–80% of the EMG rms in individual rats.

### Definition of stress susceptibility by ECG signals

The defeated rats were classified into two groups based on two variables related to cardiac activity: the arrhythmia rate and heart rate variability (HRV). The time of arrhythmia (i.e., skipping an R-peak) was automatically detected and then manually scrutinized by eye in the original ECG traces. Arrhythmia rate was counted in each 1 min. HRV is computed as the coefficient of variation of all R-R intervals observed in each 1-min bin. In Fig. 2, the Δarrhythmia rate and ΔHRV were computed as differences in the averaged arrhythmia rates and averaged HRV between the first 30 min of the post session and the last 10 min of the pre session, respectively. Rats with a Δarrhythmia rate more than 0.2 or ΔHRV more than 0.02 were classified as the susceptible group, whereas the other rats were classified as the resilient group (Fig. 2B). These criteria were set based on the observation that all 13 control rats without experiencing SD stress never exhibited such higher values, Δarrhythmia rate more than 0.2 and ΔHRV more than 0.02.

### PCA

Using PCA, three principal component dimensions were extracted from the set of correlation coefficients of LFP power changes at a specific frequency band computed from all pairs of cortical areas (e.g., Fig. 4A).

### SVM

Linear SVM was applied to define the most appropriate separation line between the susceptible and resilient groups on the dataset of correlation coefficients of LFP power changes computed from all pairs of cortical areas. To assess the degree of separation by SVM, a leave-one-out cross-validation method was applied for all points (i.e., all rats). A failure percentage was defined as a probability in which a removed point was incorrectly assigned to the group to which the point belonged. All routines were written in Matlab (MathWorks).

### Statistics

In Fig. 2D, the duration of individual behavioral patterns in the susceptible and resilient groups was compared by Student’s t-test, followed by post hoc Bonferroni correction. In Fig. 2E, the two variables in the susceptible and resilient groups were compared by Student’s t-test. In Figs 3B and 5A, LFP power at each frequency band in the susceptible and resilient groups was compared by Student’s t-test, followed by FDR correction. In Figs 4B,F,J,N,R and 5B, the difference between a pair of distributions was assessed by the Kolmogorov-Smirnov test. Correlational time changes in LFP power between groups were assessed by computing Pearson’s correlation coefficients. The null hypothesis was rejected at the *p* < 0.05 level unless otherwise specified. All data are presented as the mean ± standard error of the mean (SEM).
